# Minimally invasive mitral valve replacement for posterior leaflet tear following transcatheter edge-to-edge repair using the MitraClip system: a case report

**DOI:** 10.1093/jscr/rjaf591

**Published:** 2025-08-01

**Authors:** Hikaru Miyazaki, Ryohei Ushioda, Hidenobu Akamatsu, Tasuku Kawarabayashi, Akito Inoue, Jeonga Lee, Jun Maruoka, Yuki Setogawa, Ryo Okubo, Hiroyuki Miyamoto, Shougo Takahashi, Daisuke Takeyoshi, Shingo Kunioka, Yuya Kitani, Naoko Kawabata, Hiroyuki Kamiya

**Affiliations:** Department of Cardiac Surgery, Asahikawa Medical University, Midorigaoka 1-1-1, Asahikawa, Hokkaido 078-8510, Japan; Department of Cardiac Surgery, Asahikawa Medical University, Midorigaoka 1-1-1, Asahikawa, Hokkaido 078-8510, Japan; Department of Cardiac Surgery, Asahikawa Medical University, Midorigaoka 1-1-1, Asahikawa, Hokkaido 078-8510, Japan; Department of Cardiac Surgery, Asahikawa Medical University, Midorigaoka 1-1-1, Asahikawa, Hokkaido 078-8510, Japan; Department of Cardiac Surgery, Asahikawa Medical University, Midorigaoka 1-1-1, Asahikawa, Hokkaido 078-8510, Japan; Department of Cardiac Surgery, Asahikawa Medical University, Midorigaoka 1-1-1, Asahikawa, Hokkaido 078-8510, Japan; Department of Cardiac Surgery, Asahikawa Medical University, Midorigaoka 1-1-1, Asahikawa, Hokkaido 078-8510, Japan; Department of Cardiac Surgery, Asahikawa Medical University, Midorigaoka 1-1-1, Asahikawa, Hokkaido 078-8510, Japan; Department of Cardiac Surgery, Asahikawa Medical University, Midorigaoka 1-1-1, Asahikawa, Hokkaido 078-8510, Japan; Department of Cardiac Surgery, Asahikawa Medical University, Midorigaoka 1-1-1, Asahikawa, Hokkaido 078-8510, Japan; Department of Cardiac Surgery, Asahikawa Medical University, Midorigaoka 1-1-1, Asahikawa, Hokkaido 078-8510, Japan; Department of Cardiac Surgery, Asahikawa Medical University, Midorigaoka 1-1-1, Asahikawa, Hokkaido 078-8510, Japan; Department of Cardiac Surgery, Asahikawa Medical University, Midorigaoka 1-1-1, Asahikawa, Hokkaido 078-8510, Japan; Division of Cardiology and Nephrology, Department of Internal Medicine, Asahikawa Medical University, 2-1-1-1 Midorigaoka-Higashi, Asahikawa 078-8510, Hokkaido, Japan; Division of Cardiology and Nephrology, Department of Internal Medicine, Asahikawa Medical University, 2-1-1-1 Midorigaoka-Higashi, Asahikawa 078-8510, Hokkaido, Japan; Department of Cardiac Surgery, Asahikawa Medical University, Midorigaoka 1-1-1, Asahikawa, Hokkaido 078-8510, Japan

**Keywords:** MitraClip, minimally invasive cardiac surgery mitral valve replacement, frail patient

## Abstract

We report a case of an 80-year-old woman with severe mitral regurgitation, low ejection fraction, frailty, and acute decompensated heart failure. Due to her high surgical risk, transcatheter edge-to-edge repair using the MitraClip system (Abbott, Abbott Park, IL, USA) was attempted by the cardiology team. However, the procedure resulted in a posterior mitral leaflet tear with worsened severe mitral regurgitation. She was subsequently referred to our department, and owing to her clinical deterioration, urgent minimally invasive cardiac surgery mitral valve replacement was performed using a 29-mm bioprosthetic mitral valve (Epic; Abbott, Abbott Park, IL, USA). The patient had an uneventful recovery and was discharged on postoperative Day 13. Mitral valve surgery following failed MitraClip is considered high-risk, with elevated perioperative mortality. However, in frail patients with leaflet injury after MitraClip failure, minimally invasive cardiac surgery mitral valve replacement may represent a more appropriate and less invasive therapeutic option.

## Introduction

In elderly patients, mitral regurgitation (MR) is often associated with frailty, left ventricular dysfunction, and multiple comorbidities, making surgical intervention high risk. Consequently, transcatheter edge-to-edge repair (TEER) using the MitraClip system has become a widely accepted therapeutic option in this population [1]. However, several complications could occur with TEER. Adverse events such as single leaflet device attachment (SLDA), leaflet perforation, or leaflet tear can result in persistent or recurrent MR [2, 3], sometimes necessitating surgical bailout.

Here, we report a case of urgent minimally invasive cardiac surgery mitral valve replacement (MICS-MVR) for posterior leaflet tear following failed MitraClip implantation in a frail elderly patient with acute decompensated heart failure.

## Case report

**Figure 1:**
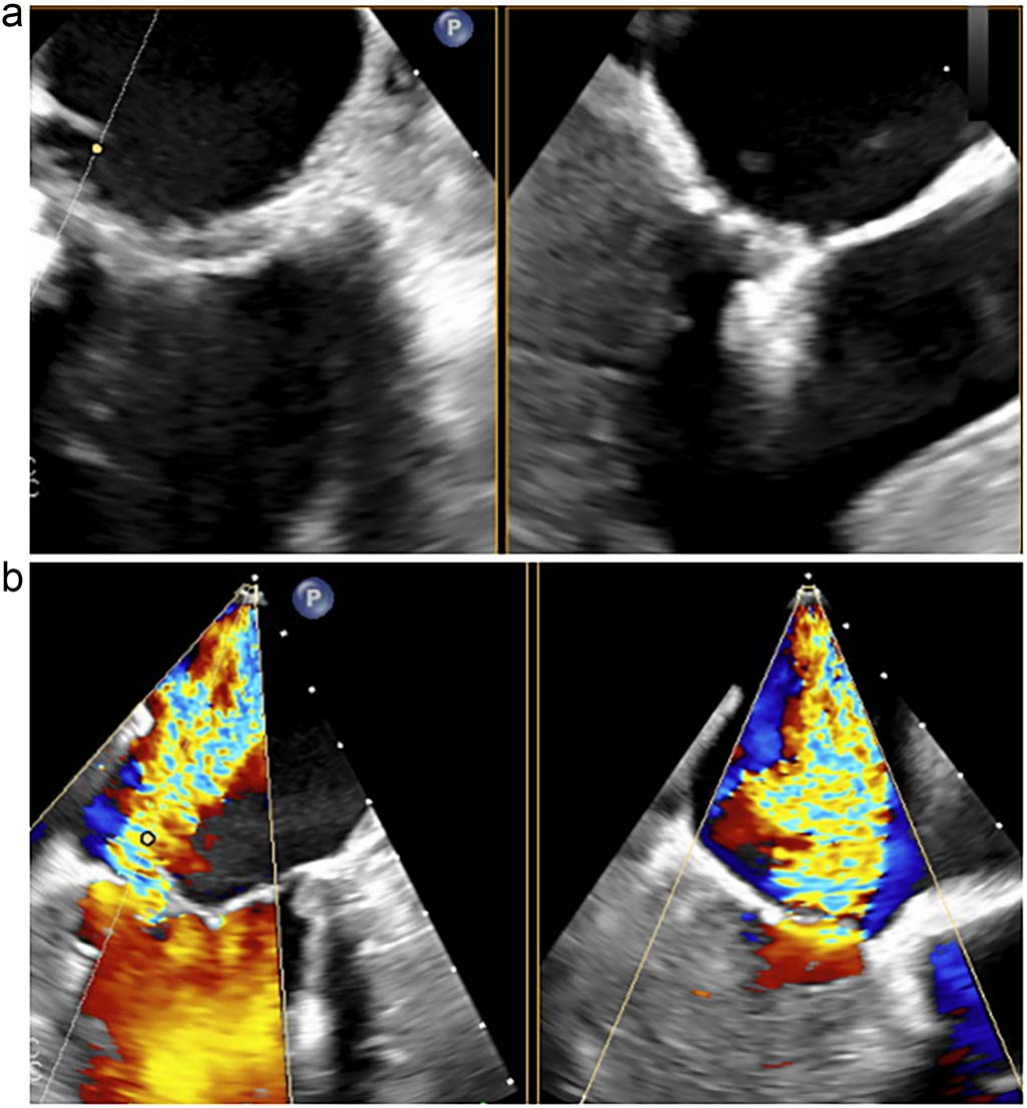
Transesophageal echocardiography following the MitraClip procedure. (a) The posterior mitral leaflet is torn, resulting in a loss of continuity. (b) Mitral regurgitation is observed originating from the site of the tear.

An 80-year-old woman with a history of chronic kidney disease, ulcerative colitis, and dyslipidemia had been followed for severe MR for the past 6 years. She presented with progressive dyspnea and was admitted with acute decompensated heart failure. Transthoracic echocardiography (TTE) demonstrated significant prolapse of the A3 segment and a markedly reduced left ventricular ejection fraction (LVEF) of 30%. Given her frailty, impaired ventricular function, and multiple comorbidities, conventional surgical mitral valve repair was deemed high risk. Therefore, the MitraClip system was selected and performed by the cardiology team. During the MitraClip procedure, however, a posterior mitral leaflet tear occurred, resulting in worsened severe MR (Fig. 1). Consequently, the patient was referred to our department for operation.

**Figure 2:**
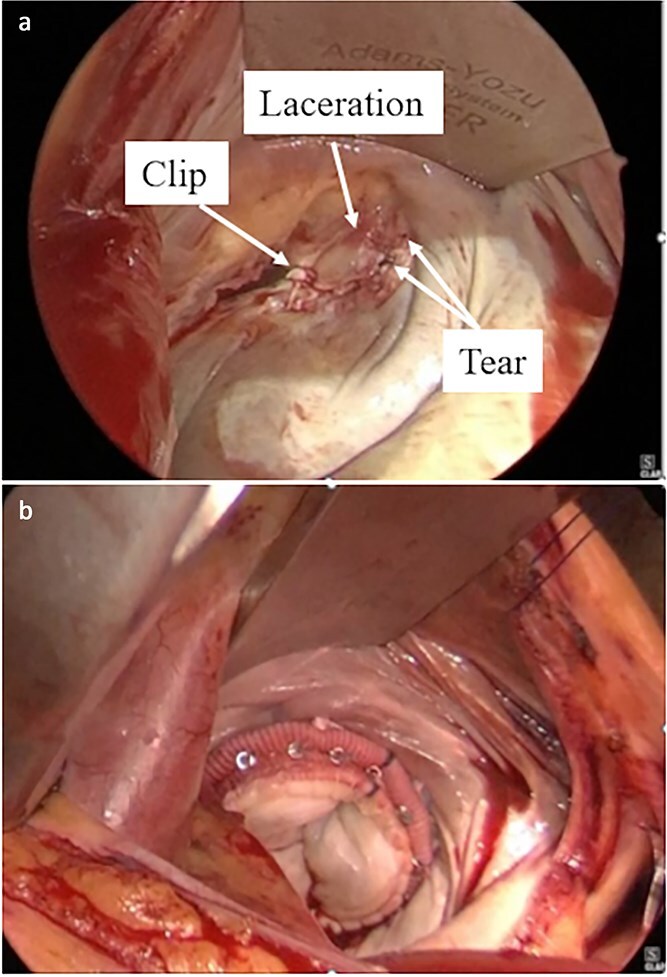
Intraoperative findings during minimally invasive mitral valve replacement. (a) Prolapse of the A3 segment and two tears in the P3 segment were observed. (b) Valve replacement was performed using a 29-mm Epic bioprosthesis.

Considering the persistent MR and the patient’s preserved general condition despite her frailty, MICS-MVR was performed via a right fourth intercostal space mini-thoracotomy. Cardiopulmonary bypass was established with femoral artery and vein cannulation. Intraoperative findings revealed a severe laceration extending from the A3 to P3 segments, with prolapse of A3 and two tears near the annulus on P3. A bioprosthetic mitral valve (Epic 29 mm; Abbott, Abbott Park, IL, USA) was implanted under direct vision (Fig. 2).

**Figure 3:**
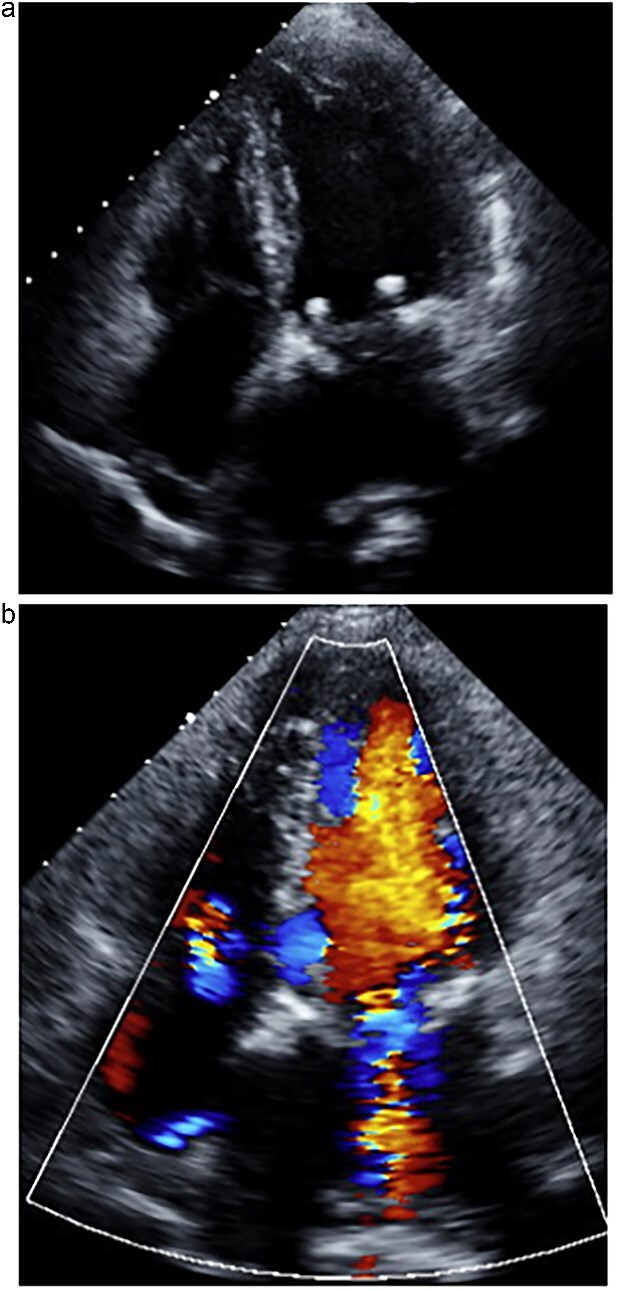
One week postoperative transthoracic echocardiography. (a) The left atrium was reduced in size. (b) Mitral regurgitation was well controlled.

The patient was extubated on postoperative day (POD) 1 and discharged home on POD 13. Postoperative TTE showed no residual MR, a LVEF of 43%, and reduced left atrial volume (Fig. 3). At 1-year follow-up, the patient remained asymptomatic with stable valve function and no recurrence of MR.

## Discussion

The MitraClip system has become an established treatment option for patients with severe MR who are considered high-risk or unsuitable for conventional surgery [1]. Although the procedure is generally safe, device-related complications such as SLDA, leaflet perforation, and leaflet tear have been reported in ~2%–6% of cases and are associated with increased morbidity and mortality [2, 3]. Among these, posterior leaflet injury is particularly concerning, as it often results in persistent MR and necessitates surgical bailout. Surgical intervention following TEER failure is technically challenging due to altered valve anatomy, advanced age, and patient frailty. Previous reports have indicated that surgical intervention after failed MitraClip implantation is associated with a substantial risk of major complications or perioperative mortality [4–6]. These findings highlight the importance of timely surgical referral and the consideration of less invasive approaches when feasible. In the present case, urgent MICS-MVR was successfully performed in a frail, elderly patient with reduced left ventricular function and multiple comorbidities. By avoiding sternotomy and limiting surgical trauma, the procedure enabled rapid MVR with an uneventful postoperative course. In cases of leaflet injury after MitraClip failure, MICS-MVR may offer an effective and practical therapeutic option, particularly for high-risk patients.
